# The crosstalk between non-coding RNAs and oxidative stress in cancer progression

**DOI:** 10.1016/j.gendis.2024.101286

**Published:** 2024-04-10

**Authors:** Qiqi Sun, Xiaoyong Lei, Xiaoyan Yang

**Affiliations:** School of Pharmaceutical Science, Hengyang Medical College, University of South China, Hengyang, Hunan 421001, China

**Keywords:** Angiogenesis, Autophagy, Cancer, Cancer energy metabolism, Epithelial mesenchymal transformation, Non-coding RNAs, Oxidative stress

## Abstract

As living standards elevate, cancers are appearing in growing numbers among younger individuals globally and these risks escalate with advancing years. One of the reasons is that instability in the cancer genome reduces the effectiveness of conventional drug treatments and chemotherapy, compared with more targeted therapies. Previous research has discovered non-coding RNAs' crucial role in shaping genetic networks involved in cancer cell growth and invasion through their influence on messenger RNA production or protein binding. Additionally, the interaction between non-coding RNAs and oxidative stress, a crucial process in cancer advancement, cannot be overlooked. Essentially, oxidative stress results from the negative effects of radicals within the body and ties directly to cancer gene expression and signaling. Therefore, this review focuses on the mechanism between non-coding RNAs and oxidative stress in cancer progression, which is conducive to finding new cancer treatment strategies.

## Introduction

**Figure 1:**
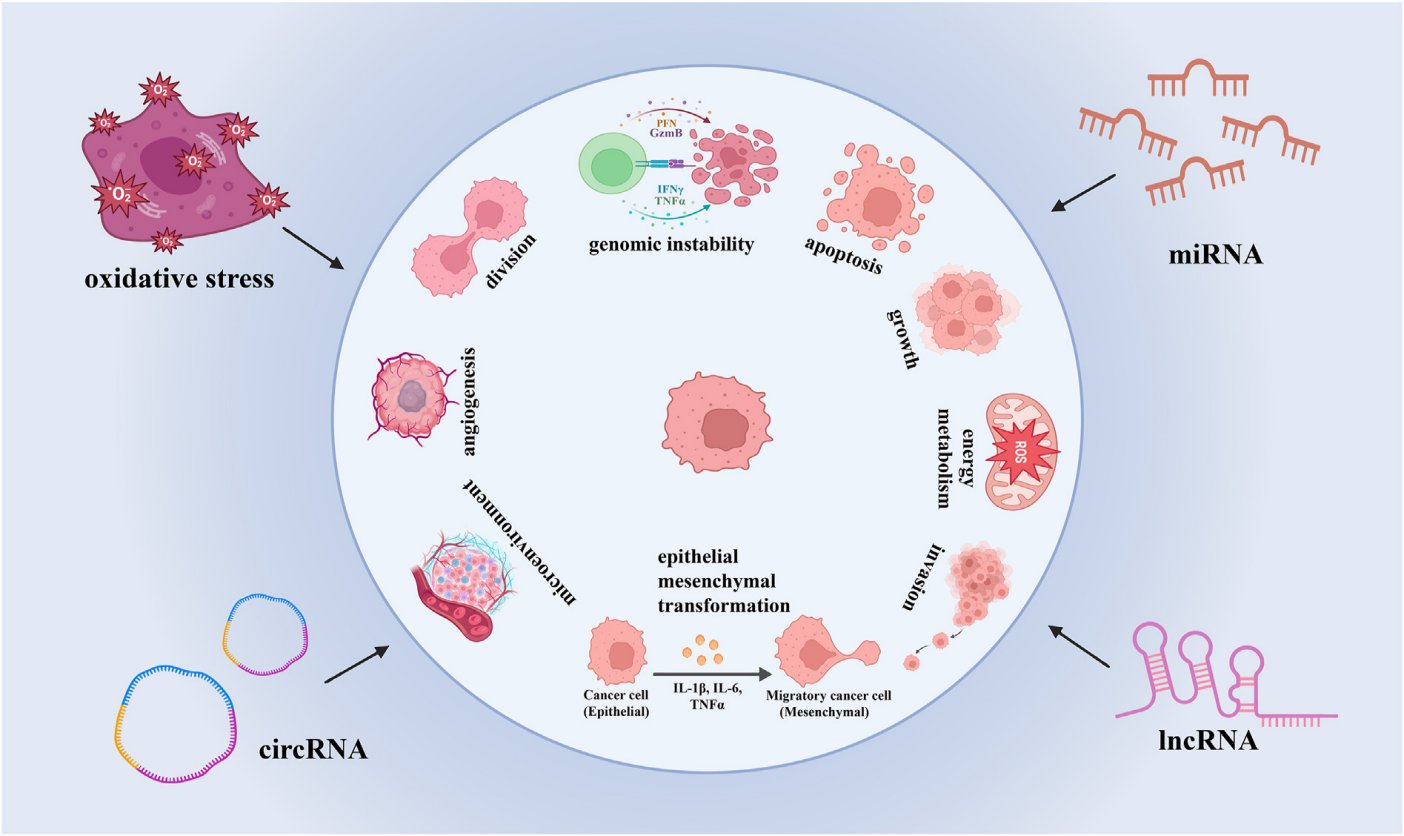
Non-coding RNAs and oxidative stress work together in various stages of cancer progression, including cancer cell growth and reproduction, cancer cell invasion, tumor microenvironment shaping, epithelial interstitial conversion, energy metabolism, and angiogenesis.

With the advancement of medical technology, cancer mortality keeps dropping, but on account of its highly heterogeneous nature, combating cancer remains challenging. According to Hanahan et al, cancer cells share several key properties, including the ability to evade growth suppressors, resist cell death, proliferate continuously and replicate immortality, and exhibit genomic instability. Additionally, they regulate cellular energy, suppress immune disruption, induce angiogenesis, activate invasion and metastasis, and promote tumor-related inflammation^1^ (Fig. 1).

Non-coding RNAs (ncRNAs) constitute a class of RNAs that do not encode proteins, comprising approximately 98% of all RNAs.^2^ They are categorized into three main groups: microRNAs (miRNAs), circular RNAs (circRNAs), and long non-coding RNAs (lncRNAs). miRNAs, with an average length of 22 nucleotides, play fundamental roles in regulating cell development, differentiation, proliferation, apoptosis, and genome stability.^3^ They also modulate the function of cancer cells and other components in the tumor microenvironment.^4^ circRNAs, characterized by a closed-loop structure without a terminating 5′ cap and 3′ poly-A tail. They are abundant, stable, and conservative in the evolutionary process.^5^, ^6^, ^7^ These characteristics make them promising cancer biomarkers in clinical diagnosis.^8^ Further, circRNAs profoundly influence gene and protein expression through transcriptional or post-transcriptional mechanisms, even impacting cell cycle progression.^9^^,^^10^ Primarily functioning as miRNA sponges, they competitively bind to mRNAs thereby disrupting miRNA-mediated post-transcriptional regulation of gene expression. lncRNAs, exceeding 200 nucleotides in length, exhibit specific expression profiles across different cell types, suggesting regulatory functions.^11^ In addition to shaping the tumor microenvironment, ncRNAs influence various cancer cell phenotypes and are increasingly recognized as valuable biomarkers in cancer diagnosis and treatment.^12^^,^^13^

Oxidative stress, characterized by the excessive production of reactive oxygen species (ROS) that overwhelms antioxidant defenses, disrupts cellular redox balance.^14^ Studies have shown that ROS are positively correlated with carcinogenesis and malignant progression of tumor cells. Oxidative stress is involved in the regulation of several biological processes, activating transcription factors such as NF-κB, AP1, p53, HIF-1α, PPARγ, Ras, Raf, β-linked protein/Wnt, p38α, VEGF, and NRF2, and regulating related enzymatic activities such as ERK1/2, PI3K/AKT, MAPK, MMP, thus significantly shaping tumor development.^15^^,^^16^ Precise regulation of ROS production and elimination in the tumor microenvironment has been strategically exploited for selective cancer targeting.^17^

ncRNAs respond to oxidative stress with changes in their cellular abundance and, in turn, regulate gene expression networks involved in ROS homeostasis and buffering.^18^ As a key step of cancer metastasis, epithelial–mesenchymal transformation (EMT) is regulated by ncRNAs, oxidative stress, and other signals, which contributes to cancer cell metastasis as well as drug resistance and correlates with poor prognosis, providing new ideas for cancer treatment.^19^ Autophagy, which is considered a lysosome degradation pathway, plays a dual role in the survival of cancer cells as ROS does, and is pivotal in both cancer cell survival and disease development, exhibiting its significance in cell homeostasis.^20^ It has been found that the dynamic process of autophagy cannot be separated from the participation of ncRNAs. Regardless of their protein-encoding abilities, these RNA molecules regulate autophagy occurrence and development via diverse transcription pathways.^21^ Likewise, their interactions are involved in developing drug resistance in cancer cells. lncRNAs were thought to be most associated with autophagy. As early as 2020, studies have shown three main axes of cancer cell metastasis, including EMT, cancer stem cells, and autophagy. Subsequently, Rohit's team detailed a therapeutic approach to limit cancer progression through autophagy and EMT regulation.^22^^,^^23^ Previous studies have focused on the interaction between ncRNAs or the regulation of cancer progression as sponges.

Despite numerous studies indicating the substantial involvement of ncRNAs in EMT and autophagy, little attention has been given to their interplay with oxidative stress. This is significantly considering the essential role of oxygen in cancer cell metabolism and its intricate interaction with ncRNAs, orchestrating enzymes and transcription factors associated with tumor development. Similarly, ROS and ncRNAs participate actively in regulating angiogenesis pathways.^24^ Nevertheless, the exact integration mechanisms remain somewhat complex. Therefore, the objective of this article is to shed light on the interaction between ncRNAs and ROS in governing cancer progression. We intend to enhance our understanding of the underlying mechanisms of cancer stress controlling while introducing innovative strategies for control. The focus will be on elucidating the crosstalk between ncRNAs and oxidative stress within the contexts of cancer EMT, autophagy, angiogenesis, and energy metabolism, spanning almost the entire course of cancer progression.

### ncRNAs and oxidative stress in EMT

EMT is essentially an evolutionarily conserved program of cellular plasticity whose gene expression is regulated directly or indirectly by EMT transcription factors such as Snail, Twist1, and ZEB1, while Wnt/β-linked protein, TGF-β/Smad, and Notch pathways are the main inducible signaling pathways. The transformation process is accompanied by alterations in stage markers (epithelial markers: E-cadherin, cytokeratin, tight junction proteins; mesenchymal markers: N-cadherin, MMP, vimentin, fibronectin, α-SMA, LLGL1/2).^25^^,^^26^ This process is orchestrated by both intra- and extracellular signaling.

In the tumor microenvironment, EMT is induced and activated by hypoxia, cytokines, and growth factors, which affect anti-tumor drug therapy. This process is triggered by the regulation of complex networks including ncRNAs.^27^ Hypoxia is involved in HIF-1α transcription through oxidative stress induced by overproduction of ROS, and the downstream effects of HIF-1α help cells adapt to hypoxia stress.^28^^,^^29^ Notably, certain ncRNAs, such as hypoxia response effectors lncRNAs, play a pivotal role in regulating hypoxic gene expression at various levels, including condensation, transcription, and post-transcriptional dynamics, serving as indirect feedback agents to HIFs or directly manipulating HIF transcription pathways. Conversely, HIFs can modulate the expression levels of lncRNAs, either directly or indirectly, utilizing the hypoxia-response elements, typically situated within its proximal promoter area.^30^ Moreover, hypoxia stimulates the activation of “central” EMT transcriptional regulatory proteins (such as Snail, Twist1, ZEB1, ZEB2, and Slug).^31^

### miRNAs and oxidative stress in EMT

**Figure 2:**
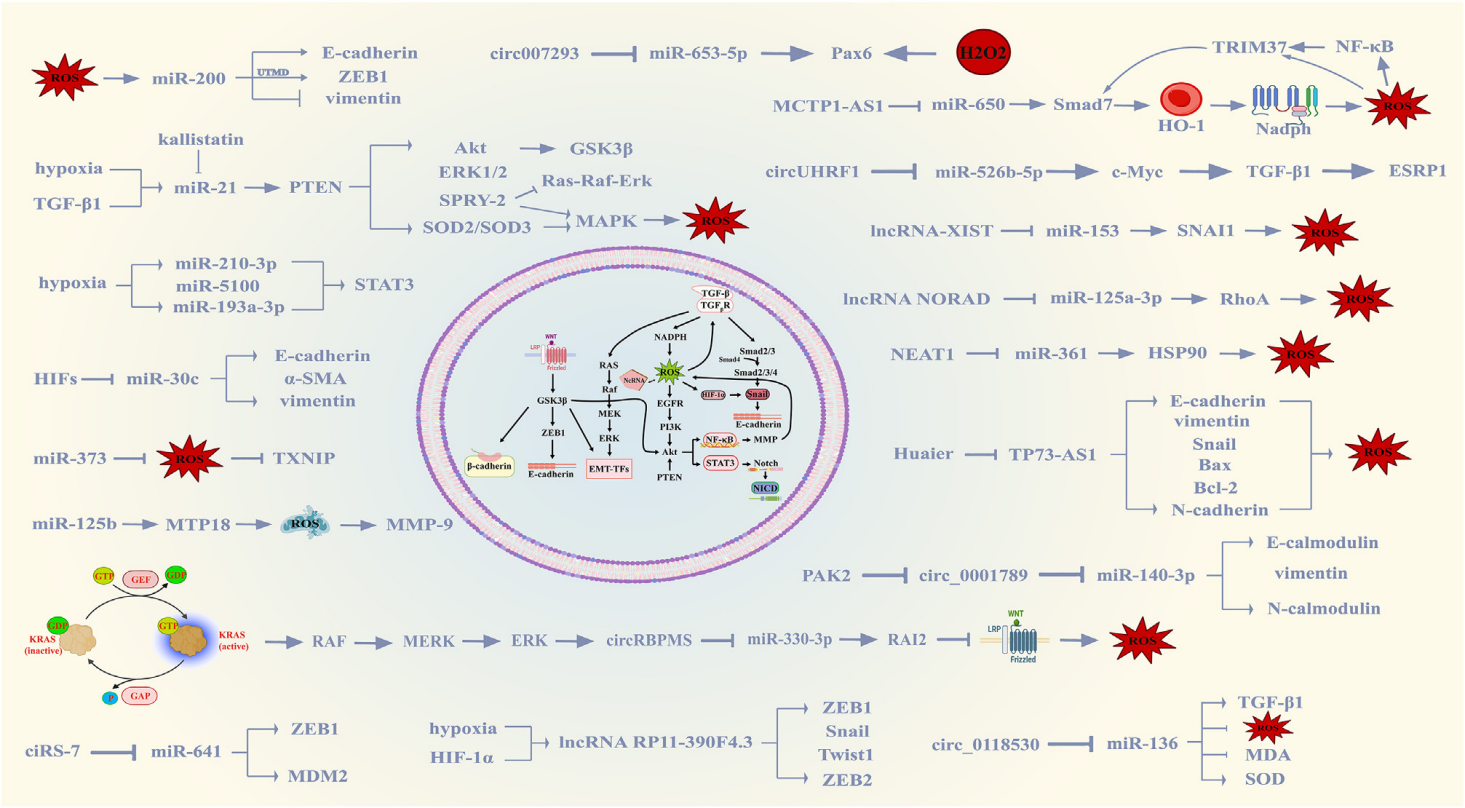
Epithelial–mesenchymal transition (EMT) denotes the process of propelling epithelial cells towards adopting a mesenchymal cell type through a distinct, phylogenetically preserved phenotypic pathway. This specific evolutionarily conserved transformation is directly controlled or significantly influenced via a host of EMT-transcription factors, notably including Snail, Twist1, and ZEB1. Furthermore, non-coding RNAs play a pivotal role within this pathway, triggering the generation of reactive oxygen species (ROS) through intricate signaling mechanisms encompassing WNT/β-catenin, TGF-β/Smad, TGF-β/non-Smad, Notch, *etc.*, interconnected with oxidative stress. Interestingly, at times, ROS themselves serve as intermediaries within these signaling cascades.

ZEB1 serves as an essential regulatory molecule pivotal to miRNA-led feed-forward loops. Its irregular expression during cancer development could potentially reinforce the EMT function, inciting self-amplifying feed-forward loops via decreased repression of its endogenous suppressors, namely, miR-141 and miR-200c (Fig. 2).^32^ Subsequent works have proven that oxidative stress imparts apoptosis and cellular aging within endothelial cells, accomplished through the up-regulation of the miR-200 gene cluster and subsequently via suppression of ZEB1 expression.^33^ miR-200 inhibition can reduce the level of E-cadherin whilst augmenting vimentin expression, which, in addition to affecting cell motility, can also lead to the acquisition of mesenchymal phenotypes, thus leading to the development of anti-cancer therapy resistance.^34^ Recently discovered ultrasound-targeted microbubble disruption-aided drug/gene delivery system effectively suppresses EMT in breast cancer cells via inducing oxidative stress and directly influencing the miR-200c/ZEB1 regulatory pathway.^35^ However, all anti-cancer drugs inevitably generate ROS during cancer cell apoptosis, and aberrant expression levels of all miR-200 family members may help regulate the drug resistance process under oxidative stress. Considering that cancer cells can adapt to environmental changes,^36^ further exploration of the EMT-ROS-miR-200 pathway holds promise for attenuating early cancer metastasis and advancing strategies to conquer cancer drug resistance. Its powerful function determines that it is significantly differentiated from oxidative stress in tumor progression.

miR-21, which is overexpressed in various cancers, is induced by TGF-β1 or hypoxia and influences EMT by regulating the transcription of invasive markers such as E-cadherin via the PTEN/AKT/GSK3β pathway.^37^^,^^38^ The antagonism of miR-21 has previously been found to reverse EMT and cancer stem cell phenotypes by targeting PTEN, inactivating AKT and ERK1/2 pathways in breast cancer.^39^ In addition, miR-21 can directly or indirectly down-regulate SOD2/3 and SPRY2 (a negative regulator of Ras-Raf-ERK signaling) to stimulate MAPK-mediated ROS production, thus promoting tumorigenesis.^40^ The endogenous protein kallistatin can inhibit oxidative stress-mediated EMT by silencing miR-21, thus achieving the role of tumor prevention.^41^

Hsp70s are ubiquitous molecular chaperones involved in cellular defense mechanisms. Under oxidative stress, their expression increases as part of the cellular protective response, essential for cell survival under adverse conditions.^42^^,^^43^ The application of miR-223 mimics and inhibitors on the rabbit glaucoma model demonstrated that miR-223, specifically targeting the Hsp70s within retinal ganglion cells, could suppress cell multiplication, orchestrate apoptosis, and stimulate inflammation.^44^ Regulation of Hsp70 depends on the sequences of miR-85 and Hsp70 3′ untranslated region, containing the miRNA target site.^45^ Hsp70s inhibit EMT in mesenchymal cells primarily by negatively regulating ROS production and phosphorylation and nuclear translocation of Smad3 and Smad4.^46^ With numerous miRNAs capable of impacting ROS generation and inhibiting EMT via their regulation of Hsp70s, it is conceivable that a strategy focusing on heat shock protein inhibition could play an integral role in cancer treatment.

Studies on the co-effects of miRNA and oxidative stress in cancer cell EMT are extensive, and there are many others involved. For example, the metastasis of miR-193a-3p, miR-210-3p, and miR-5100 as hypoxic bone marrow-derived mesenchymal stem cell-derived exosomes may promote cancer cell invasion by activating STAT3 signal-induced EMT.^47^ Similarly, it has been demonstrated that a process driven by HIFs, where miR-30c expression is down-regulated in renal cell carcinoma cells, promotes EMT modifications in slug stages by influencing E-cadherin and modifying α-SMA and vimentin expression.^48^ miR-373 reduces thioredoxin interacting protein expression by interacting with 3′ untranslated region of thioredoxin-binding proteins to reduce thioredoxin interacting protein-dependent ROS and activate EMT.^49^ miR-125b exhibited a diminished presence within hepatocellular carcinoma tissues producing an up-regulation of mitochondrial translocator protein 18 kDa, leading to mitochondrial fission. Subsequent mitochondrial damage resulted in elevated levels of ROS that further enhanced matrix MMP-9 expression, hence providing a direct impetus for EMT and tumor metastasis.^50^

### lncRNAs and oxidative stress in EMT

The interaction between lncRNAs and oxidative stress also plays a role in regulating the EMT process. For instance, lncRNA RP11-390F4.3, which is directly regulated by hypoxia/HIF-1α, activates the expression of multiple EMT regulators, including Snail, Twist 1, ZEB1, and ZEB2.^51^ Among numerous molecules, Huaier's anti-tumor efficacy was established by suppressing the transcription of TP73-AS1, leading to concurrent changes in the expression of EMT-associated proteins (E-cadherin, Snail, N-cadherin) and vimentin and mitochondrial proteins (Bax and Bcl-2). Subsequently, an increase in ROS levels was recorded.^52^ This finding was confirmed in cholangiocarcinoma, but the exact association between them requires further investigation. When knockdown experiments were conducted on lncRNA CYTOR in oral squamous cell carcinoma, its binding to RNA-binding protein HNRNPC was found to promote mitochondrial respiration and glycolysis, thereby altering ROS levels. This process is achieved by positively regulating the expression of ZEB1,^53^ linking EMT to energy metabolism, and offering new perspectives for cancer treatment. Studies on endometrial cancer have shown that lncRNA MCTP1-AS1 regulates endometrial cancer cell proliferation, migration, invasion, and EMT processes through the miR-650/Smad7 axis.^54^ Currently, Smad7 is known to be a downstream target of TRIM37. Smad7 can affect the expression of NF-κB by activating HO-1 to regulate NADPH-mediated ROS production. However, ROS and NF-κB can also act as upstream regulators to regulate TRIM37 and inhibit this process.^55^, ^56^, ^57^ As an E3 ubiquitin ligase exhibiting in mulibrey nanism,^58^ TRIM37 plays a crucial role in the malignant potential of tumors through its overexpression.

Adding layers of complexity to the existing scenario, lncRNAs may augment the EMT phase in cancerous cells due to their influence on miRNA expression, exhibiting synergy with oxidative stress. For example, in osteosarcoma, lncRNA-XIST promotes oxidative stress-induced osteosarcoma cell invasion, migration, and EMT through sponge-directed down-regulation of miR-153 and its direct messenger RNA target Snail1.^59^ This represents the first exploration of the lncRNA-XIST/miR-153/Snail1 pathway, recognized as a pivotal node of oncogenesis. In addition, elevated lncRNA NORAD expression, which has been shown to be associated with poor prognosis in pancreatic cancer, promotes EMT under hypoxia-induced conditions by competing with miR-125a-3p to regulate the expression of the small GTP-binding protein RhoA.^60^ NEAT1, highly expressed in cervical cancer, competitively binds to miR-361 and inhibits its expression to indirectly up-regulate Hsp90 expression, thereby inducing changes in the levels of EMT-related molecules and spheroid formation.^61^ Hsp90 is a molecular chaperone whose expression correlates with oxidative stress and participates in the regulation of homeostasis. Cold plasma cancer cell therapy exploits this mechanism by driving ROS to trigger cleavage of Hsp90 chaperones and degradation of PKD2, which further stabilizes HIF-1α and activates NF-κB and VEGFA, and subsequent tissue vascularization to promote tumor angiogenesis.^62^, ^63^, ^64^ This reveals that despite targeting the same protein, distinct mechanisms govern cancerous cell progression.

### circRNAs and oxidative stress in EMT

To date, circRNAs associated with oxidative stress have been found to influence EMT always by directly targeting miRNAs, a mechanism mirrored by the drugs involved. For instance, exposure to ropivacaine in glioma cells induces oxidative stress and apoptosis, leading to the up-regulation of E-calmodulin expression and inhibition of vimentin protein expression through the circSCAF11/miR-145-5p axis.^65^ Similarly, up-regulation of circRBPMS in breast cancer affects cancer progression by directly targeting the miR-330-3p/retinoic acid-induced 2 axis, in which EMT and KRAS/ERK pathways are involved in circRBPMS overexpression.^66^ Follow-up studies have identified retinoic acid-induced 2 as a novel antagonist of the Wnt/β-linked protein signaling pathway, thereby regulating oxidative stress.^67^ In oral squamous cell carcinoma, circUHRF1 promotes EMT and tumor growth through the circUHRF1/miR-526b-5p/c-Myc/TGF-β1/ESRP1 feedback loop.^68^ Furthermore, following the observation that hydrogen peroxide levels affect Pax6 expression and subcellular localization,^69^ Qiuyu Lin et al found that exosomal circ007293 induced EMT and enhanced invasion and migration of thyroid cancer cells via the miR-653-5p/Pax6 axis.^70^ However, whether the association with oxidative stress is coincidental or causative needs further investigation. Functionally, ciRS-7 up-regulates the expression of ZEB1 and MDM2 by sponging miR-641, thereby contributing to EMT in ovarian cancer cells.^71^ Dual-targeted therapies may be a new idea for blocking this pathway. The deletion of hsa_circ_0118530 in the human granulosa-like tumor cell line KGN was found to use TGF-β1 to induce EMT, coinciding with suppressed ROS accumulation and malondialdehyde levels, induced SOD activity, with miR-136 being its direct target.^72^ Additionally, circ_0001789 has the property of negatively regulating miR-140-3p, and PAK2 overexpression can reverse the changes in EMT protein markers caused by it, which is related to the malignant phenotype of gastric cancer, and could serve as a biomarker for poor prognosis.^73^

### ncRNAs and oxidative stress in autophagy

Autophagy, an evolutionarily conserved catabolic mechanism, provides cells with sustainable biomolecules and energy sources to maintain homeostasis under stressful conditions like tumor microenvironment.^74^ The protein kinase TOR exhibits distinct evolutionary stabilization properties. ROS and their inhibitory effects on mitochondrial function may trigger mitochondrial autophagy in a specific manner, with the mTORC1 being central. Regarding how the growth factor signaling of mTORC1 is regulated, it mainly involves the important channel IGF-1-PI3K-AKT, which negatively regulates autophagy induction. Interestingly, ATG1 assembly intensifies autophagy by restraining mTORC1 dynamics, thus achieving an optimal equilibrium between autophagy enactment and cellular growth restriction.^75^

In genetically engineered mouse models, autophagy has been shown to limit tumor initiation by regulating DNA damage and oxidative stress.^76^ As ROS levels increase, they actively stimulate and enhance the initiation of autophagy, a process tightly linked to stress signaling pathways. Specifically, the cysteine protease ATG4 is inhibited in the cell due to the accumulation of ROS. As a result, ATG4 is unable to perform its function properly, leading to the accumulation of ethanolamine phosphate precursors, which are prerequisites for the initiation of autophagosome formation.^77^ There is growing evidence that autophagy can be regulated by manipulating the transcription of ATGs and post-transcriptional regulatory ncRNAs.^78^

In conclusion, ncRNAs and oxidative stress can interact with autophagy to participate in regulating the cancer process. However, the effectiveness of inhibition of autophagy in clinical practice remains relatively limited. Therefore, the method of inhibiting or enhancing autophagy should be selected based on the specific circumstances to address the issue most effectively.^79^

### miRNAs and oxidative stress in autophagy

As a major regulator of hypoxia response, HIFs have been shown to promote the transcription of key autophagy proteins, including BNIP3 and NIX.^80^ BNIP3, being a proapoptotic gene, has exhibited potential as a universal cancer suppressor in several types of cancer, such as prostate cancer,^81^ esophageal squamous cell carcinoma,^82^ and pancreatic cancer.^83^ At the same time, BNIP3 influences cancer progression as a target for various ncRNAs. As early as 2010, TP53-mediated tumor suppressor miR-145 was found to inhibit prostate cancer by negatively regulating BNIP3 by targeting its 3′ untranslated region.^81^ Further studies by Zengxi Xin et al have shown that inhibition of the PI3K/AKT signaling pathway promotes apoptosis and oxidative stress by overexpression of miR-145-5p in tongue squamous cell carcinoma.^84^ As a key regulator of autophagy, therapeutic strategies targeting PI3K/AKT/mTOR play an important role in enhancing tumor chemosensitivity and avoiding drug resistance and are expected to be used in the treatment of pancreatic cancer, lung cancer, and hepatocellular carcinoma.^85^, ^86^, ^87^, ^88^, ^89^ In addition, miR-145 protects follicular somatic granulosa cells from oxidative stress-induced apoptosis by targeting KLF4 in the H_2_O_2_-induced *in vitro* model and 3-NP-induced *in vivo* ovarian oxidative stress model to maintain normal ovarian function.^90^ In breast cancer cell lines, miR-145 directly interferes with the Ang2 gene translation and its expression negatively correlates with its promoter methylation level, leading to the inhibition of migration and invasion of breast cancer cells.^91^ Although miR-145 plays a role as a biomarker in a variety of cancers, its regulatory mechanism network is complex due to its versatility, necessitating further research for its clinical application.

Apart from miR-145, numerous other miRNAs interact with oxidative stress to affect autophagy. For example, in dysfunctional endothelial cells, reduced miR-155 has been found to reduce oxidation-induced damage, promoting cell proliferation through the up-regulation of autophagy, and thus affect the expression of the autophagy-related gene ATG5.^92^ Studies have shown that miR-383 induces apoptosis and mitochondrial ROS production by down-regulating PRDX3 in human glioma cells U87,^93^ thereby promoting oxidative stress-induced autophagy. Moreover, overexpression of miR-27a in breast cancer cells not only attenuates mammosphere formation and survival but also chemosensitizes breast cancer cells by down-regulating glutathione-related NRF2, CTH, and SLC7A11, leading to attenuation of ROS defense system and autophagy.^94^ Transfection of miR-506-3p resulted in inhibition of apoptosis and intracellular ROS production in pancreatic ductal adenocarcinoma cells by CK2α and promotion of cell proliferation and inhibition of ROS-induced up-regulation of PTGR2 and showed profound autophagy induction.^95^ It plays a similar role in lung cancer, where miR-506-3p causes elevated ROS levels through negative regulation of NF-κB p65 expression, leading to increased p53 activation and apoptosis in lung cancer cells.^96^ In laryngeal squamous cell carcinoma cell lines JHU-SCC-011 and HNO210, the methyl donor S-adenosylmethionine was found to induce ER stress and autophagy, with ROS production being downstream of the ER stress response, accompanied by down-regulation of miR-888-5p.^97^

Indeed, under certain circumstances, autophagy plays an indirect regulatory role in miRNAs. For instance, miR-101 can activate erastin- and RSL3-induced iron death by targeting the glutamate-cysteine ligase. Overexpression of STMN1 in its direct targets of regulated autophagy-related proteins (RAB5A, ATG4D, and STMN1) relieves miR-101-mediated cell autophagy inhibition. This theory has been shown to enhance the sensitivity of hepatocellular carcinoma cells to cisplatin while sensitizing breast cancer cells to 4-hydroxytamoxifen-mediated cell death.^98^^,^^99^ Gene therapy targeting the miR-101/autophagy/ROS pathway holds the potential for advancing chemotherapeutic processes, reinforcing the link between miRNA, autophagy, and ROS. Wang et al found that sodium butyrate inhibits bladder cancer cell migration and induces AMPK-mTOR pathway-dependent autophagy and ROS via the miR-139-5p/Bmi-1 pathway, while overproduction promotes sodium butyrate-induced cysteine-dependent apoptosis and autophagy.^100^

### lncRNAs and oxidative stress in autophagy

lncRNAs can simultaneously target ATG genes, and as vital autophagy regulators, their expression can also be modulated by autophagy. This property may provide new targets for cancer treatment.^101^

One of the earliest discovered lncRNAs, H19,^102^ interferes with almost all processes that contribute to chemoresistance. Mechanically, H19 can exert its carcinogenic effects by activating autophagy, inhibiting apoptosis, and enhancing EMT, thus increasing the aggressiveness of cancer cells.^103^ By assessing autophagy flux levels, the study of metformin found that its powerful tumor-inhibiting ability may be thwarted by down-regulating H19-mediated ROS production to inhibit autophagy, thus impeding cancer cell vitality.^104^ While the precise mechanism remains unclear, these findings reinforce the intimate link between H19, oxidative stress, and autophagy. Comparative experiments have shown that blocking the MAPK/ERK signaling pathway to induce osteosarcoma is one mechanism by which H19 affects its chemotherapy resistance and oxidative stress. In addition, inhibiting the PI3K-AKT-mTOR pathway to up-regulate autophagy is another mechanism through which it plays a role in hepatocellular carcinoma cell apoptosis.^105^^,^^106^ In glioblastoma, lncRNA MDHDH promotes the degradation of MDH2 by mediating its binding to the proteasome subunit PSMA1 after generation, thereby reducing the NAD^+^/NADH ratio of cells, affecting energy supply and inducing autophagy through the AMPK/mTOR pathway, thus exerting tumor-inhibitory effects.^107^ Pyrroline-5-carboxylate reductase 1, while linked with mitochondrial oxidative stress and ROS generation,^108^ is up-regulated by antisense lncRNA-RP11-498C9.133, which leveraging ROS for triggering mitochondrial autophagy results in breast cancer progression.^109^

lncRNA loc146880 connects autophagy, ROS, and lung cancer. Internalization of PM2.5 (a class of particulate matters, <2.5 μm in diameter) increases the expression of lncRNA loc146880 in a manner that induces ROS production, thereby promoting autophagy.^110^

Deletion of lncRNA EGFR-AS1 in cervical cancer cells promotes autophagy-mediated iron death through down-regulation of miR-133b-mediated EGFR expression, accompanied by an increase in ROS levels and a decrease in cell viability. In contrast, overexpression of EGFR can weaken or even reverse the material changes that occur in this process.^111^

Besides, lncRNAs can impact autophagy by indirectly sequestering miRNA. lncRNA SCAMP1 regulates ZEB1/JUN and autophagy via miR-429 to promote renal cell carcinoma in children under oxidative stress.^112^ Xu Z et al discovered that lnc-NLC1-C, overexpressed in glioma cells, targets the process where miR-383 reverses mitochondrial ROS inhibition induced by PRDX3 overexpression, promotes autophagy and oxidative stress, and enhances apoptosis in cancer cells.^113^^,^^114^

### circRNAs and oxidative stress in autophagy

circCDR1as, acts as a sponge for miR-671-5p in oral squamous cell carcinoma, leading to enhanced autophagy, and increased cytotoxicity by regulating lysosomal activity, the AKT/ERK/mTOR signaling pathway, and ROS. Notably, this effect is significantly amplified in hypoxic microenvironment.^115^ PAK2 can be activated by hyperosmolarity and oxidative stress through increased binding to the endoplasmic reticulum, playing an important role in stress signaling.^116^ PAK2 is significantly up-regulated in neuroblastoma and its expression can be significantly reduced by miR-195. circ_0013401 overexpression has a targeting effect on this process, which in turn prevents apoptosis and autophagy.^117^ Furthermore, H_2_O_2_ treatment inhibits osteosarcoma progression, and circKMT2D knockdown suppresses this outcome via the miR-210/autophagy pathway.^118^ Moreover, concerning drug action, modulation of the circ_0001345/miR-106b/ATG16L1 axis expression may be a potential protective mechanism of cyanidin-3-glucoside against hepatocellular carcinoma.^119^

Furthermore, several profound studies have analyzed the influence of drug resistance. Based on the finding that ATG7-dependent autophagy protects breast cancer cells from mitogen-induced oxidative stress, Qiuli Wang et al demonstrated that hsa_circ_0092276 promotes doxorubicin resistance in breast cancer cells by regulating autophagy through the miR-348/ATG7 axis.^120^^,^^121^ Similarly, the activation of ATG7 by down-regulating miR-3657 in apatinib regulates autophagy, while silencing circRACGAP1 directly targets this pathway to increase the sensitivity of gastric cancer cells to apatinib.^122^ circHIPK3 promotes oxaliplatin resistance in colorectal cancer by sequestering miR-637, which is dependent on the inhibition of autophagy-associated cell death through the STAT3/Bcl-2/beclin1 signaling pathway.^123^

circRNAs can also act alone with oxidative stress in autophagy. For example, circTBC1D14, which is significantly up-regulated in triple-negative breast cancer, physically interacts with fused in sarcoma, altering its subcellular localization through protein arginine methyltransferase 1-associated transfer of fused in sarcoma. It balances stress granule formation and autophagy to maintain cellular homeostasis under hypoxic conditions.^124^ Overexpression of circRNA-104075 counteracts the PI3K/AKT and Wnt-β-catenin pathways inhibited by matrine in glioma cell line U251. Furthermore, matrine induces autophagy and apoptosis by regulating the circRNA-104075/Bcl-9 axis.^125^ These findings further validate the significant role of specifically targeted circRNA pathways in mediating drug effects.

### ncRNAs and oxidative stress in angiogenesis

Angiogenesis is the physiological process involved in the formation of blood vessels from pre-existing ones.^126^ The first step in angiogenesis involves the role of angiogenic stimuli,^127^ such as hypoxia or inflammation. Its mechanism is mainly regulated by chemical stimuli such as VEGF, Ang, HGF, HIF, IGF, TGF-β, and TNF.^126^ Of great interest here are the roles of VEGF-dependent angiogenesis coupled with HIF signaling, a pathway often perturbed by ROS.^128^

The interconnection between the HIF-1α/ROS pathway initiates tissue-specific angiogenesis via enhanced expression of VEGF, along with its cognate receptors VEGFR1 and VEGFR2. Conversely, VEGF potently stimulates ROS generation within endothelial cells by facilitating the activity of NADPH oxidase.^129^ Previous evidence suggests that concurrent occurrence of activators such as TGF-β1 within the evolving tumorous tissue and hypoxic regions could synergistically stimulate VEGF expression, thereby facilitating a suitable milieu for tumor angiogenesis. Interestingly, Ras-altered MME cells respond to VEGF by triggering neighboring endothelial cells to express the higher-affinity signaling receptor VEGFR2. Subsequently, these cells become highly sensitive to VEGF.^130^ Notably, Ras mutation stabilizes HIF-1α and escalates the level of VEGF-A transcription activity. Moreover, an activated Ras state correlates with augmented ROS generation.^131^ ROS also affects VEGF-induced dimerization and autophosphorylation of VEGFR2, which are indispensable for VEGFR2 activation and consequent angiogenesis.^132^ Additionally, ROS stabilizes HIF-1α, whereby the sulfhydryl group at Cys326 of PHD2 is oxidized by ROS, leading to impaired heterodimerization, allowing for the abnormal accumulation of HIF-1α and subsequent nuclear translocation.^133^ ncRNAs, in turn, regulate the expression of these two key proteins interacting with oxidative stress to influence angiogenesis.

Consequently, the discussion subsequently turns towards those ncRNAs participating in oxidative stress interactions during the progression of angiogenesis.

### miRNAs and oxidative stress in angiogenesis

**Figure 3:**
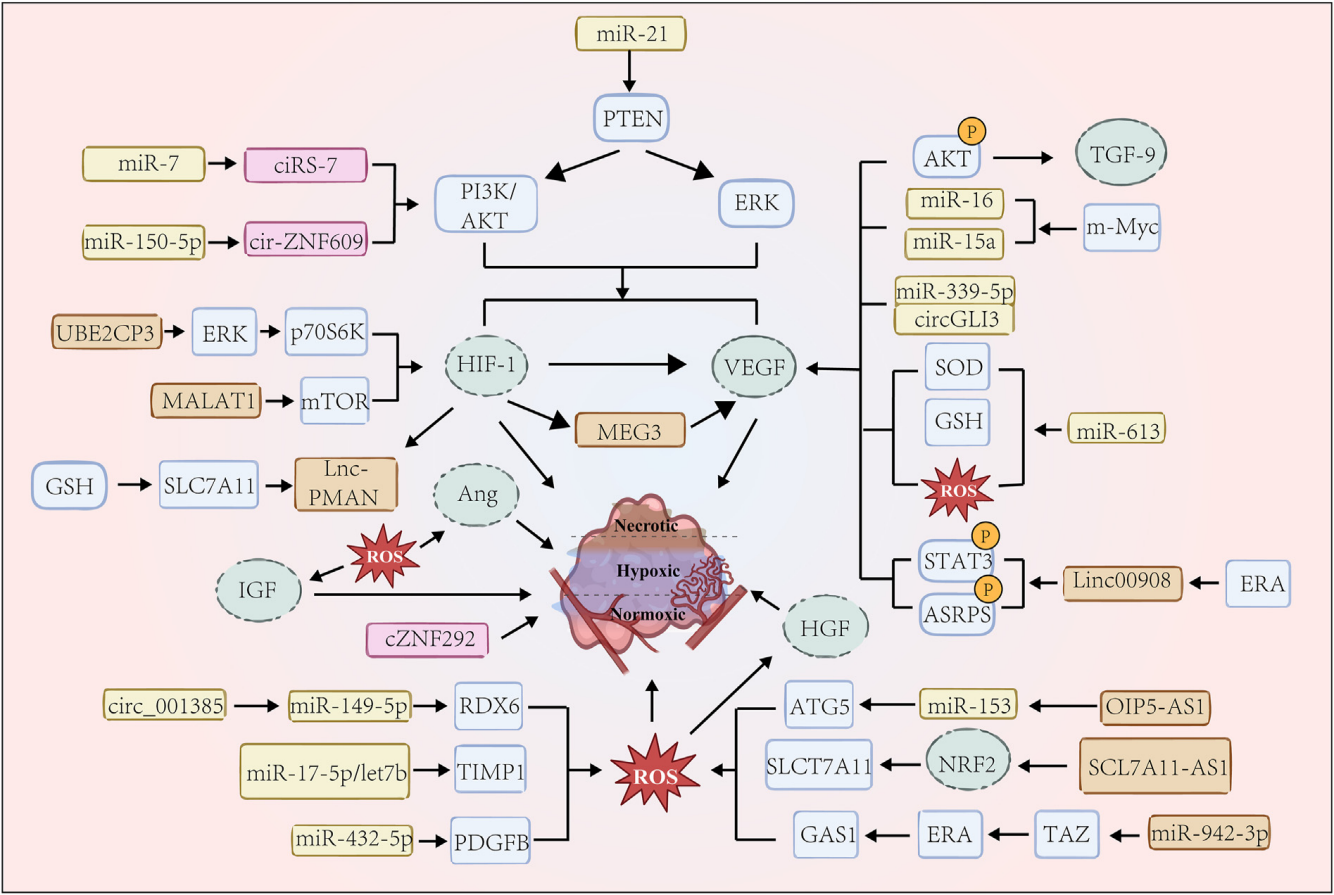
The regulation of angiogenesis is directly under the umbrella of various stimuli such as VEGF, Ang, HIF, IGF, and TGF-β, with VEGF emerging as the most significant player in this context. The role of non-coding RNAs in regulating angiogenesis is intriguing; they specifically target the generation of aforementioned angiogenic agonists (cZNF292 notwithstanding). During this intricate targeting process, reactive oxygen species (ROS) invariably serve as downstream signal mediators influencing the function of other critical factors or directly impacting angiogenesis which presents multiple levels of regulatory process.

A series of transfection experiments and comparisons have confirmed that miR-21 stimulates tumor angiogenesis by targeting HIF-1 and VEGF while simultaneously activating AKT and ERK downstream parallel pathways (Fig. 3).^134^ This idea has been validated in studies of ovarian cancer cells and is also associated with paclitaxel resistance regulation.^135^ In doxorubicin treatment of cell drug-resistant breast cancer cells, miR-21 can also act by targeting PTEN.^136^ Low levels of miR-4732-3p suggest early cardiotoxicity after anthracycline therapy in breast cancer patients, which exerts cardioprotective mechanisms through the TGF-β and Hippo signaling cascades, partially triggering angiogenic and antioxidant responses.^137^

Platelet-derived growth factor B secreted by OS cells is down-regulated by highly expressed miR-432-5p to regulate angiogenesis.^138^ It is crucial for cell survival under high oxidative stress^139^ and presents a potential therapeutic target for osteosarcoma by targeting the crosstalk between the miR-432-5p/platelet-derived growth factor-B signaling pathway and oxidative stress.

A positive feedback loop between the transcription activator zone with PDZ-binding motif and miR-942-3p, which is highly expressed in breast cancer tissues, acts on angiogenesis, EMT, and glucose metabolism through GAS1 while regulating ROS levels.^140^ This underscores the versatility of a single miRNA and the multifaceted use of a single cancer regulator for clinical intervention.

Hypoxia-induced miR-15a/16 is directly controlled by oncogenic signaling molecule c-Myc, contributing to multiple myeloma angiogenesis via specific targeting VEGF-A and primarily correlates with advanced cancer stages.^141^^,^^142^ An investigation into curcumin's influence on colorectal cancer cell lines unveiled that administration with curcuminoids suppressed the development of colon tumors by deregulating the ROS/miR-27a/Sp pathway, serving as an inhibitor of VEGF signaling.^143^ A similar mechanism has been observed in gastric cancer.^144^ As for gastric cancer resistance, miR-613 prevents NUTM2A-AS1-induced resistance of gastric cancer cells to bitter ginseng alkaloids, a process achieved by affecting oxidative stress-related ROS production, glutathione levels, and SOD activity, subsequently adjusting the expression levels of VEGFA.^145^

miR-17-5p and let7b influence angiogenesis by regulating tissue inhibitor 1 of metalloproteinases,^146^ while high expression of MT1-MMP in prostate cancer cells can induce ROS, which may occur dependently or independently of extracellular matrix adhesion.^147^ Previous studies have linked these two miRNAs to the prognosis of prostate cancer patients and tumor metastasis, suggesting a possible pathway of action.

### lncRNAs and oxidative stress in angiogenesis

MALAT1 is highly expressed in the hypoxic environment of angiogenesis.^148^ On the basis that the abnormally high expression of MALAT1 in osteosarcoma was found to be similar to the high expression in hypoxia induction, by injecting osteosarcoma tumor transplanted mice with siMALAT solution, Zhang et al found that MALAT1 in osteosarcoma cells induced up-regulation of pro-angiogenic factors, including VEGF-A mainly through MALAT1/mTOR/HIF-1α pathway, and HIF-1α showed positive feedback effect and selectivity. It did not affect normal tissue-induced angiogenesis.^149^

In triple-negative breast cancer, estrogen receptor α-regulated LINC00908 down-regulates VEGF expression by influencing the phosphorylation process of ASRPS and STAT3 to affect tumor angiogenesis.^150^ Interestingly, STAT3 is activated by moderate levels of ROS, and mitochondrial STAT3 phosphorylation enhances murine 4T1 breast cancer cell development by increasing the complex I conjugated system and decreasing ROS levels.^151^

As one of the top ten most abundant lncRNAs in human umbilical vein endothelial cells, MEG3 promoter activity can be enhanced by hypoxia treatment and HIF-1α overexpression in response to VEGFR2 expression, regulating the angiogenesis process. It did not affect VEGFR1, Notch1, DLL4, and Hes1 gene expression.^152^ Notably, MEG3 expression varies with oxidative stress levels. In the xenotransplantation model, MEG3 overexpression promotes angiogenesis of breast cancer cells, while decreased phosphorylated AKT levels inhibit downstream angiogenesis-related factors VEGFA and TGF-β9.^153^ AKT-mediated phosphorylation affects the processing and stability of mitochondrial calcium uniporter 1, leading to the production of ROS involved in tumor progression.^154^ The biological role of MEG3 in endothelial cells is beyond doubt.

UBE2CP3 has been shown to promote hepatocyte VEGFA secretion into the tumor microenvironment and enhance tumor cell-induced angiogenesis through activation of the ERK/p70S6K/HIF-1α pathway,^155^ while ERK signaling has previously been noted to play a role in the regulation of tumor EMT.^156^ The exact mechanism underlying the relationship between EMT and angiogenesis is unclear, but what is known is that ncRNA has a communicative role in oxidative stress. High expression of exosomal lncRNA OIP5-AS1 in osteosarcoma enhances ATG5 expression by suppressing miR-153, promoting cellular autophagy.^157^ Interestingly, ATG5, rather than acting directly on endothelial cells, interferes with ROS-dependent signal transduction pathways for optimal regulation of angiogenesis,^158^ intricately linking oxidative stress with lncRNA/miRNA pathways, autophagy, and angiogenesis.

lncRNA-PMAN exhibits high expression in gastric cancer's peritoneal metastases, being up-regulated by HIF-1α. It enhances the stability of SLC7A11 (membrane channel transporter protein) mRNA by promoting the cytoplasmic distribution of ELAVL1, whose accumulation increased glutathione levels and inhibited the accumulation of ROS and iron in gastric cancer cells, leading to further proliferation and development of tumor cells.^159^ As an adjacent sense transcript of SLC7A11, lncRNA SLC7A11-AS1 in colorectal cancer may contribute to the removal of excess ROS through up-regulation of the NRF2/SLC7A11 antioxidant system,^160^ and SLC7A11-AS1 has previously been found to reduce ROS levels by stabilizing NRF2 in pancreatic cancer.^161^ Although promising as a cancer therapeutic target, clinical studies are still lacking in this area.

### circRNAs and oxidative stress in angiogenesis

As mentioned above, circRNAs are stable and conserved and play a major role in tumor angiogenesis either by acting as targeting sponges for miRNAs or competing as endogenous RNA molecules.^162^^,^^163^ Recently, studies have identified circZNF609 as a sponge for miR-150-5p, directly regulating AKT3 expression.^164^ Additionally, circ_0001789, previously associated with cancer malignancy, has been found to enhance the angiogenic potential of endothelial cells.^73^ Zhi-xin Li et al studied small intestinal epithelial cells (IPEC-J2) and found that circGLI3 regulates VEGFA by directly binding to miR-339-5p, altering the levels of glutathione peroxidase and inflammatory factors, thereby reducing the damage caused by oxidative stress.^165^ Furthermore, circ_0011385 increased PRDX6 expression by conserving miR-149-5p in cervical cancer cells, promoting angiogenesis and malignant behavior.^166^ Another example is ciRS-7, one of the earliest circRNAs discovered,^167^ which acts as a miR-7 sponge to influence the PI3k/AKT pathway in hepatocellular carcinoma angiogenesis, usually accompanied by miR-41 level changes.^168^^,^^169^ This suggests that the circRNA network may contain multiple sponges. However, some circRNAs act by other mechanisms. For example, cZNF292, which down-regulates angiogenesis in glioma cells, has been found to be induced by hypoxia in a time-dependent manner and is independent of HIF-1α, showing the regulation of angiogenesis in hepatoma cells. However, it has been found that it cannot act as an RNA sponge, and the mechanism remains ambiguous.^170^^,^^171^

In conclusion, the mechanism of circRNAs in tumor angiogenesis is intricate, involving various ncRNAs in complex regulatory networks.

### ncRNAs and oxidative stress in cancer energy metabolism

The activation of growth signal transduction, driver gene activation, and mTOR activation are all contingent upon ATP phosphorylation. This fundamental aspect of cancer cell metabolism has been underscored by three contemporary metabolic hypotheses: the renowned “classic Warburg effect”, the emerging “cancer cell symbiosis”, and the novel “glutaminolysis”. These hypotheses collectively emphasize the pivotal role of energy metabolism in facilitating the survival and proliferation of malignant cells.^172^ Studies have shown that ncRNAs contribute to the redistribution of tumor metabolism. Mechanically, this regulatory influence can either directly impact the expression levels of mRNAs and proteins involved in metabolism or mediate through related factors that control the synthesis of these enzymes.^173^ The production of ROS is inevitable in energy metabolism, with a direct correlation between metabolic rate and ROS generation. In particular circumstances, this may induce a disturbance in the oxidative status, culminating in cellular oxidative stress.^174^ Tumor cell metabolism is reprogrammed from oxidative phosphorylation to aerobic glycolysis even when oxygen is sufficient, a phenomenon known as the “Warburg effect”. Enhanced Warburg effect helps cancer cells minimize oxidative stress.^175^^,^^176^ Here, we mainly discuss the role of non-coding RNAs and oxidative stress in the most common “Warburg effect”.

### miRNAs and oxidative stress in cancer energy metabolism

To meet higher metabolic requirements, miRNAs regulate various enzymes and protein molecules that are easily reprogrammed in cancer cells.^177^

**Figure 4:**
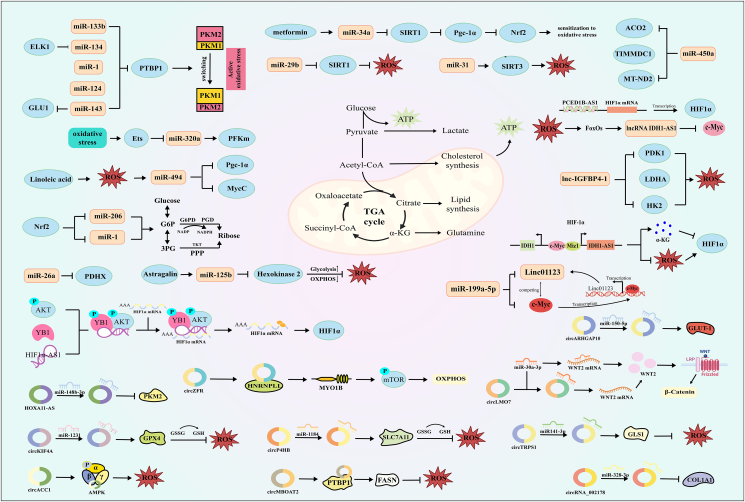
The energy metabolism permeates virtually every facet of a cancer cell's lifecycle. NcRNAs exhibit a direct influence on the expression patterns of mRNAs and proteins implicated in metabolism, such as PKM and G6PD. Additionally, these molecules manipulate the production of pivotal enzymes and associated factors, such as PTBP and c-Myc, thereby impacting diverse metabolic pathways that culminate in the generation of reactive oxygen species (ROS) linked to oxidative stress. Occasionally, this can induce iron deprivation culminating in cell demise.

The Warburg effect, characterized by the enhancement of the glycolytic pathway in cancer cells, focuses on PKM1/2 as its key molecules. Evidence suggests that aberrant miRNA targeting PTBP1 disrupts the cancer-specific energy metabolism across diverse types of cancer cells through PKM2 up-regulation, a phenomenon widely observed in carcinogenesis (Fig. 4).^178^ This affects the production of ROS and associated ATP, interfering with cancer resistance. One notable example is that ELK1 promotes aerobic glycolysis and enhances chemical resistance to osteosarcoma *in vitro* through a miR-134/PTBP1 signaling cascade.^179^ This concept was also verified by Sugiyama et al in gastric cancer cells, where miR-133b switches the expression of PKM subtype from PKM2 to PKM1 by silencing PTBP1, a process in which ROS production is partly due to the transition from glycolysis to oxidative phosphorylation and contribution to autophagy induction.^180^ Similarly, ectopic expression of miR-143 in renal cell carcinoma triggers disturbances in glucose metabolism by reducing GLUT1 and PTBP1/PKM axis expression, shifting glycolysis towards oxidative phosphorylation, and stimulating autophagy by increasing ROS levels.^181^ The enhanced oxidative stress of miR-124 in colorectal cancer cells and the ectopic expression of the miR-124/PTB1/PKM1/PKM2 axis constitute a feedback cascade, inducing apoptosis and autophagy. These elements act as tumor suppressors and metabolic regulators.^182^ It is noteworthy that miR-26a specifically targets a protein essential for colorectal cancer regulation known as pyruvate dehydrogenase X,^183^ as observed with PTBP1-associated miR-1 and miR-133b in colorectal tumors.^184^

In addition to PKM, other pathways may influence the Warburg effect. Down-regulated miR-320a was found to contribute to the disuse of septum and up-regulated rate-limiting glycolytic enzyme PFKm in response to oxidative stress in lung cancer and cultured muscle cells. Proto-oncogenic transcription factor Ets protein also regulates PFKm through this mechanism.^185^ Targeting miR-320 is not limited to a specific glycolytic pathway and may serve as a new target for blocking further malignant tumorigenesis. In ovarian cancer cells, miR-450a targets aconitase 2, suppresses TIMMDC1 and MT-ND2, acts on the citric acid cycle, and affects glutamine metabolism and mitochondrial activity,^186^ resulting in the accumulation of oxygen in the cell, sparking oxidative stress that exacerbates the outcome. Sirtuins are key to NAD-dependent deacetylase activity,^187^ with differential localization correlating with different functions. Reduced SIRT1 expression attenuated ROS production in H_2_O_2_-treated epithelial ovarian cancer cells, attributed to negative regulation of SIRT1 targeting by miR-29b.^188^ Similarly, miR-31-SIRT3 impairs mitochondrial membrane potential and structural integrity, and dysregulation of this axis contributes to the generation of oxidative stress in oral squamous cell carcinoma.^189^ Remarkably, miR-34a can be induced to inhibit the SIRT1/Pgc-1α/NRF2 pathway by the diabetic drug metformin, thereby increasing the sensitivity of wild-type p53 cancer cells to oxidative stress and therapeutic reagents.^190^ Additionally, sustained activation of NRF2 signaling in lung cancer cells disrupted the expression of miR-1 and miR-206, directing carbon fluxes towards the pentose phosphate pathway and the tricarboxylic acid cycle, thereby bolstering cellular redox homeostasis via augmented NADPH synthesis.^191^ G6PD is activated upon acute exposure to ROS, diverting glucose breakdown from glycolysis through the oxidative branch of the pentose phosphate pathway into nucleotide biosynthesis, thereby causing an elevation of the conversion of NADP to NADPH. This latter reaction serves as a pivotal mechanism for alleviating oxidative stress levels.^192^ This regulatory process represents a novel link between miRNA regulation, glucose metabolism, and ROS homeostasis in cancer cells. Linoleic acid affects mitochondrial biogenesis through ROS-induced miR-494 expression and inhibition of its target genes MYCC and Pgc-1α, leading to reduced energy production and quiescence in colorectal cancer.^193^ Administration of astragalin's cytotoxic properties to hepatocellular carcinoma cells stifled HK2 expression, and redirected glycolysis towards oxidative phosphorylation, yielding ROS accumulation and growth stasis. Subsequent studies attribute this effect to a miR-125b elevation.^194^

### lncRNAs and oxidative stress in cancer energy metabolism

lncRNAs mediate post-translational modifications of aerobic glycolytic-related enzymes, such as HIF-1α, c-Myc, and NF-κB transcription factors, and indirectly affect the activity of metabolism-related proteins through AMPK, PI3K/AKT, and p53.^195^

In lung cancer tissues, lnc-IGFBP4-1 has been implicated in affecting ATP production levels and the expression of aerobic glycolytic-related enzymes, including HK2, PDK1, and LDHA.^196^ There are a variety of transcriptional regulatory elements in the structure of LDHA, such as HIF-1, MYC, KLF4, and FOXM1,^197^ among which HIF-1, FOXM1, and KLF4 have been verified to be related to the regulation of ROS signal transduction and play an important role in shaping oxidative stress conditions.^198^ Recent findings suggest that Linc01123, activated by c-Myc in non-small cell lung cancer tissue, promotes aerobic glycolysis in cancer cells through miR-199a-5p/c-Myc sponge mechanism, during which HK2 and LDHA levels and activities are improved.^199^ Similarly, lncRNA IDH1-AS1, a c-Myc transcriptional target, catalyzes homodimerization of the enzyme IDH1, thereby augmenting its enzymic action, subsequently leading to increased α-KG and reduced ROS generation. However, while the former result promotes HIF-1α expression, the latter does the opposite.^200^ This discovery uncovers two complementary yet potentially overlapping mechanisms governing HIF-1α expression and glycolytic functionality. Studies on kidney cancer cells under sugar starvation have shown that energy stress regulates FILNC1/AUF1 subcellular localization through FOXOs. The interaction between FILNC1 and AUF1 leads to the down-regulation of c-Myc protein levels, thereby enhancing glucose uptake and lactic acid production.^201^ This effect has also been applied to the prognosis of cancers such as lung adenocarcinoma. Specifically, HOXA11-AS controls PKM2 through miR-148b-3p, promoting the proliferation and glycolytic activity of lung adenocarcinoma cells, with its overexpression predicting an unfavorable outcome.^202^ Furthermore, PCED1B-AS1, identified as a pro-oncogenic lncRNA in glioblastoma, acts in a HIF-1α-dependent manner to promote the Warburg effect, but it is unclear whether there is the regulation of feed-forward loop formation to amplify this effect.^203^ By leveraging the established role of HIF1A-AS1 (the inherent antisense RNA counterpart of HIF-1α mRNA) in modulating HIF-1α′s oxidative response under conditions of oxidative stress within human induced pluripotent stem cell-derived endothelial cells,^204^ Fengyu Xu et al have discovered that HIF-1α can interact with the hypoxia-response elements of HIF1A-AS1 promoter, subsequently amplifying glycolysis and facilitating the resilience of pancreatic cancer towards gemcitabine through the AKT/YB1/HIF-1α cascade.^205^ Moreover, lncRNA CYTOR, already mentioned above, is also involved in tumor progression by affecting energy metabolism.^53^ These examples underscore the significant impact of lncRNAs on energy metabolism pathways through their interactions with enzymes and regulatory factors, highlighting the importance of considering their interactions with miRNAs.

### circRNAs and oxidative stress in cancer energy metabolism

In breast cancer cells, silencing circRNA_002178 reduces oxygen consumption rate and extracellular acidification rate while reducing ATP/AMP, ATP/ADP, and NADH/NAD ratios. This effect on energy metabolism is inhibited by miR-328-3p. In turn, the expression of COL1A1 downstream can also be induced to decrease by circRNA_002178, thus inhibiting energy metabolism.^206^ Notably, the decline in oxygen consumption rate and extracellular acidification rate often acts as a physiological indicator of mitochondrial oxidative stress.^207^ circNFATC3 is implicated in regulating invasion, migration, mitochondrial function, and oxidative phosphorylation in breast and ovarian cancer cells through multiple pathways, including STAT3, mitochondrial malfunction, axon guidance, pentose phosphate pathway, PTEN signaling, IL15 production, SIRT signaling, and NF-κB signaling. This regulation occurs via interaction with members of the Let-7 family of miRNAs and binding to multiple binding sites of RNA-binding proteins.^208^ In non-small cell lung cancer, diminished expression of circARHGAP10 obstructs proliferation and migration by targeting the miR-150-5p/GLUT1 axis. However, GLUT1 overexpression has minimal impact on miR-150-5p concentration.^209^ Recently, identified as a novel biomarker for early diagnosis of lung adenocarcinoma, circP4HB protects lung adenocarcinoma from iron death by regulating miR-1184/SLC7A11 axis-mediated glutathione synthesis.^210^ circKIF4A was also found to be able to directly sponge miR-1231 by up-regulating the expression of the antioxidant protein GPX4 to inhibit iron death and promote thyroid-like cancer progression.^211^ circLMO7, significantly up-regulated in gastric cancer, acts as an miR-30a-3p sponge to influence glutamine metabolism through the Wnt2/β-catenin regulatory pathway.^212^ Not coincidentally, exosome-derived circTRPS1 from bladder cancer-derived cells can regulate intracellular ROS homeostasis and CD8^+^ T-cell depletion via the miR-141-3p/GLS1 axis, with GLS1-mediated glutamine metabolism involved in circTRPS1-mediated alterations.^213^

circRNAs in energy metabolism can also function through miRNA-independent mechanisms. For example, recent investigations have shown circZFR to be a novel regulator of oxidative phosphorylation adaptation to energy stress. It regulates selective splicing such as MYO1B by stabilizing HNRNPLL proteins, promoting oxidative phosphorylation and cellular proliferation, thereby stimulating the AKT-mTOR signaling route.^214^ Interestingly, under metabolic stress, circACC1 was observed to stimulate PFK2 activity and glycolysis, while also expediting fatty acid β-oxidation through enhanced activation of the AMPK complex.^215^ circMBOAT2 enhances PTBP1 stability by protecting it from ubiquitin/proteasome-dependent degradation, which in turn promotes cytoplasmic export of FASN mRNA, leading to ultimately reprogramming lipid metabolism and alterations in redox homeostasis.^216^

### ncRNAs and oxidative stress in cellular growth and proliferation

Mutation impaction upon RTK signaling frequently culminates in cellular transformation, evident among numerous forms of malignancies, and can be acknowledged as upstream growth detectors. These mutations affect either RTKs or downstream pathway constituents like MAPK and PI3K/AKT, thereby inciting augmented cell proliferation, survival, invasiveness, and metastatic potential.^217^ Prior investigations have indicated that diminished levels of ROS stimulate the expansion and multiplication of malignant cells. Furthermore, subsequent research has disclosed that cancer-derived IgG contributes to lower intracellular ROS generation via diminishing overall cellular antioxidant potential, culminating in the promotion of cancer cell development and proliferation through the MAPK/ERK signaling cascade.^218^

Moreover, ROS demonstrates a substantial correlation with cellular proliferation-related signaling pathways, particularly in the context of drug therapy. Metformin exhibits notable efficacy beyond its role in managing diabetes, in which ncRNAs are also involved. It possesses the capability to suppress cellular proliferation and migration via a mechanism involving reduced activity within the mTOR signaling pathway. This unusual mechanism involves suppression of the PI3K/AKT/mTOR signaling cascade as well as activation of an inhibitory protein known as REDD1. Furthermore, it elicits a G0/G1 phase cell cycle arrest, accompanied by reduced expression of c-Myc and down-regulation of IGF1R. These antiproliferative effects are mediated by AMPK activation and increased ROS production.^219^ Moreover, metformin's correlation with SOD within breast cancer indicates a potential ability to restrict tumor growth via triggering apoptosis utilizing the mitochondrial pathway. Episodes of this process are paralleled by the variations observed in pro-apoptotic Bax and anti-apoptotic Bcl-2, MMP-2, MMP-9, miR-21, and miR-155 expression.^220^

### miRNAs and oxidative stress in cellular growth and proliferation

FOXO activity hinges upon precise post-translational modifications. Notably, ROS-activated MAPKs, particularly the ROS-induced c-Jun N-terminal kinase, enhance FOXO function through diverse mechanisms. These include directly phosphorylating Thr295 or Thr317 in FOXO4, as well as modifying and restraining cytoplasmic 14-3-3 proteins, which sequester FOXO, and indirect inhibition of AKT activity. Further, oxidative stress can induce a loss of FOXO activity via the nuclear factor kappa-light chain enhancer of NF-κB pathway. Exposure to oxidative stress triggers up-regulation of NF-κB signaling, resulting in phosphorylation and suppression of FOXO3α, mediated by IkB kinase.^221^ FOXO is a key signaling protein in the growth factor pathway downstream of AKT.^222^ K-Ras promotes pancreatic cancer cell proliferation by up-regulating miR-155 expression through the MAPK and NF-κB pathways, inhibiting the FOXO3a transcription factor, and subsequently inhibiting the expression of two major antioxidants, SOD2 and catalase, to increase ROS levels.^223^ Elevated miR-155 levels appear to stimulate lung cancer cell proliferation and expand the G2/M cell population by hindering FOXO1 function, which consequently heightens ROS generation. Interestingly, these effects seemed to be mitigated when administering N-acetylcysteine.^224^ Astonishingly, butyrate stimulates programmed cell death and impairs cellular proliferation via elevated miR-22 production. This enhances the repression of SIRT1, causing a decrease in SOD function, thereby augmenting ROS. This mechanism occurs via the PTEN/p-AKT signaling cascade.^225^ miR-99a significantly blocks the NOX4/ROS/Akt signaling pathway, effectively reducing the amount and activity of MMP2 and MMP9, two important matrix metalloproteinases,^226^ ultimately decreasing the invasion and metastatic ability of lung adenocarcinoma cells.^227^

Phosphorylation of Bim is a decisive step for proliferating cell survival. Abrogation of Bim phosphorylation during mitosis could potentially provoke apoptosis spanning from metaphase to gap phase one, while phosphate addition by MEK/ERK to Bim attenuates the pro-apoptotic signature of the protein, creating a connection between cell progression and survival pathways.^228^ It is established that miR-128a suppresses the proliferation of medulloblastoma cancer cells by augmenting ROS levels via meticulous suppression of Bmi-1.^229^ Within colorectal cancer cells, amplification of miR-210 was observed to augment ROS production and suppress clonogenicity and proliferation concurrently. This regulation was observed in the restriction of cell division into the G2/M phase. Furthermore, miR-210 is instrumental in inducing apoptosis, a process linked to amplified expression of the pro-apoptotic protein Bim and potentiated activity of caspase 2.^230^

Treatment with LPS, a potent activator of TLR4 in human primary lung carcinoma cells, boosts the formation of ROS. This subsequently stimulates miR-21 expression, culminating in enhanced tumor progression.^231^ As previously documented, miR-21 effectively manages oxidative stress during cancer via intricate signaling networks, influencing both oxidative and antioxidant enzyme expression. Occasionally, this control mechanism appears to be reversed, potentially contributing to therapeutic strategies.^37^, ^38^, ^39^, ^40^, ^41^^,^^134^, ^135^, ^136^ The revealing data indicates a sophisticated interplay between ROS production and miR-21 abundance. A recently concluded study discovered that diminished expression of PDCD4 stimulates the PI3K/AKT pathway.^232^ Divergent expression of miR-21-5p culminates in augmented quantities of PDCD4, a crucial player in mediating excessive ROS production after acute Cr (VI) exposure. This up-regulated protein is instrumental in guiding apoptosis and eliciting growth suppression within cells.^233^ The pro-oncogenic activity of specificity protein makes it an example of cancer cell addiction to non-oncogenes. Notably, the ROS-dependent targeting of the specificity protein transcription factors constitutes an essential pathway contributing towards the curative potential of ROS-inducing medications.^234^ ZBTB stands as a transcriptional repressor that competes for binding within copious GC-rich cis-elements and dislodges the specificity protein transcription factors, consequently diminishing the manifestation of genes under specificity protein regulation.^235^ The underlying mechanism to explain the suppression of colon cancer cell proliferation by the nonsteroidal anti-inflammatory drug GT-094 critically involves mitochondrial destabilization and the consequent initiation of ROS. These processes subsequently result in a ROS-regulated disconnection between miR-27a and the ZBTB10-specificity protein regulatory axis.^236^ Intriguingly, this functional mechanism has also been observed with regard to various compounds that suppress cancer cell expansion, such as sulindac sulfide, curcumin along with its synthetic cyclohexanone and piperidine analogs, and piperlongumine.^234^^,^^237^^,^^238^

### lncRNAs and oxidative stress in cellular growth and proliferation

Influencing cancer cell cycle progression is a major feature of lncRNA-independent action. As an integral lncRNA that is remarkably lowly expressed in non-small cell lung cancer tissue, TUG1 serves as a growth modulator via direct regulation by p53. Concurrently, it epigenetically influences HOXB7 expression partly through interaction with PRC2, sparking activation of the AKT and MAPK pathways, thereby stimulating cellular proliferation.^239^ Another study has shown that deoxyelephantopin proficiently suppresses the propagation of uterine leiomyoma cells by inducing a cell cycle arrest at the G2/M transition phase, subsequently triggering apoptosis initiated by ROS and executed via caspase-3 within the mitochondria. It also dampens the up-regulation of oncogenic lncRNAs, including H19, HATOIR, BANCR, and Linc-ROR expression in tumor cells.^240^ In an extensive analysis of 54 distinctly aberrant lncRNAs involved in hepatocellular carcinoma progression, a noteworthy expression pattern was observed for the lncRNA ZFSA1. This lncRNA can stimulate the rise of intracellular ROS levels by down-regulating essential molecules such as NADH dehydrogenase proteins (*e.g.*, NDUFA6, NDUFB4, and NDUFB11), ultimately facilitating cancer cell proliferation and migration.^241^ Further studies have determined that down-regulation of lncRNA GAS5 enhances malignant melanoma cell proliferation by influencing the transition from the G1 to S phase of the cell cycle via a rise in the expressions of cell cycle regulatory proteins including D1, CDK4, and p27. Concurrently, suppression of GAS5 augments programmed cell death through an upsurge in Bcl-2 expression. The silencing of GAS5 also increases concentrations of superoxide anion, NADPH, and oxidized glutathione by up-regulating the expression of both NOX4 and G6PD, coupled with elevated NOX activity.^242^ Significantly, MACC1-AS1, identified as a stress-responsive gene, regulates the abundance of MACC1 via direct interaction with its mRNA sequence, stimulation of AMPK, and displacement of Lin28. Additionally, it enhances metabolic flexibility by elevating the expression of GLUT1, HK2, and LDHA, resulting in amplified glycolytic and antioxidant processes, fostering gastric cancer cell migration and invasion, with minimal impact on EMT regulation.^243^

In some cases, lncRNAs also compete with miRNAs to regulate the proliferation process of cancer cells. CBR3-AS1 in non-small cell lung cancer may act as a competing endogenous RNA, and its down-regulation through miR-409-3p expression reduces SOD1 expression, increases γH2AX formation, raises ROS levels, reduces proliferation, invasion, and migration, inhibits cell cycle progression, and promotes apoptosis.^244^ Based on numerous studies, H_2_ stands out for its remarkable potential to augment health conditions through the mitigation of detrimental ROS.^245^ For instance, implementing the consumption of H-enriched water during radiation therapy for individuals diagnosed with malignant hepatocellular carcinoma proved to markedly lower blood hydrogen peroxide concentrations while enhancing biological antioxidant functionality,^246^ leading to a considerable enhancement in overall quality of life. Interestingly, it was observed that the presence of hydrogen holds an inhibitory effect on the proliferation and migration of gastric cancer cells, primarily by orchestrating the regulation of lncRNA MALAT1. In this context, both lncRNA MALAT1 and miR-124-3p mutually repress one other's expression, thereby inhibiting EZH2 gene expression altogether.^247^

### circRNAs and oxidative stress in cellular growth and proliferation

FOXK1 is an important regulator of cell proliferation, quiescence, and differentiation in stem cell populations and cancers. Specifically, FOXK1 invigorates the class I PI3K/AKT/mTOR cascade while suppressing class III PI3K, with consequential impacts on the malignant behavior of gastric cancer.^248^ circPPP2R4 has been shown to reduce ROS production by sponging miR-646 to up-regulate FOXK1 expression, and its overexpression promoted colorectal cancer cell proliferation, migration, and invasion.^249^ Circular RANGAP1 silencing significantly suppresses lung cancer cell proliferation and migration via modulation of the miR-512-5p/SOD2 axis, as demonstrated by Chunhua Zhao et al.^250^ circBNC2 up-regulates GNAS expression in a miR-142-3p-dependent manner and inhibits oral squamous cell carcinoma cell growth but induces apoptosis and oxidative stress.^251^ The role of oxidative stress and circRNAs in the proliferative process of cancer cells is inseparable from apoptosis.

Saliently, SLC7A11 constitutes an integral component of the glutamate cystine antiporter Xc^−^ system, which actively stimulates the influx of cystine and catalyzes glutathione synthesis, thus effectively counteracting oxidative stress and the emergence of iron-induced cell death.^252^ Contemporary investigations have unveiled that SLC7A11 modulates the MAPK pathway, specifically promoting p38 activation while inhibiting extracellular signal-regulated kinase in nasopharyngeal carcinomas.^253^ Additionally, SLC7A11 possesses inherent functional autonomy as a glutathione-independent redox system by sustaining the redox cycling indispensable for balanced signaling and communication at the periphery of the plasma membrane.^254^ For the first time, researchers have revealed a circular RNA associated with iron death in cervical cancer lesions specifically: circEPSTI1. By silencing circEPSTI1 and targeting the miR-375/409-3P/515-5p-SLC7A11 pathway, the study succeeded in reducing the relative ratio of glutathione to reduced glutathione in cervical cancer cells through a competing endogenous RNA-mediated manner, significantly reduced the ratio of glutathione to GSSG in cervical cancer cells, and inhibited the expression of GPX4 protein. This series of changes led to a massive accumulation of lipid peroxidation products in the cell membrane layer, which in turn triggered a lipid peroxidation event that induced the formation of the iron death phenomenon, which in turn further contributed to the division and growth of cervical cancer cells.^255^ circ_0067934/miR-545-3p/SLC7A11 signaling attenuates iron death in thyroid cancer cells, and regulation of this pathway may be a potential therapeutic target.^256^ The silencing of circFNDC3B, via regulatory mechanisms involving miR-520d-5p, directly impacts the observed suppression of GPX4 and SLC7A11 expression alongside an elevation in ROS, iron, and Fe^2+^ concentrations within oral squamous cell carcinoma cells. This modulatory effect significantly impairs cell proliferation yet potently stimulates apoptosis rates in these cancerous cells.^257^ Not coincidentally, the circ_0016142/miR-188-3p/GPX4 axis also proliferated in the same manner in hepatocellular carcinoma cells.^258^ circTTBK2, through its regulatory function on cellular proliferation, invasive potential, and iron-induced apoptosis, culminates in the repression of ITGB8 expression via miRNA sponge mediation of miR-761 in glioma cells.^259^ Low levels of circ_0000190 ominously forecast an unfavorable outcome for gastric cancer patients. More specifically, acting as a suppressor of miR-382-5p, it amplifies the regulation of ZNRF3, thereby stimulating gastric cancer cell proliferation, and migration, and thwarting the function of erastin or RSL3. As a consequential effect, this inhibition mediates iron demise.^260^

## Conclusion

In the feedback loop of cancer, lncRNA acts as the main regulatory factor, while circRNA mainly functions as a miRNA sponge and interacts with RNA-binding proteins to regulate gene transcription and translation. These three ncRNAs all impact ROS creation and pathway. circRNAs have been less reported in this regard than the other two types, but attention has been paid to their relationship with innate immune regulation.^261^ Their potential as an entry point for cancer treatment is enormous. ncRNAs and oxidative stress always play a role in the invasion and migration of cancer cells through ROS generation, affecting the formation of blood vessels and changing metabolic pathways. Notably, these impacts mostly apply to malignant tumors. Most of the research is in the preliminary theoretical stage. Although the findings in this area relate to the whole process of cancer development, including prognosis, oxidative stress and ncRNA studies in reducing cancer incidence have been less involved in benign tumors and cancer prevention. At the same time, little or no research has been conducted on alleviating suffering in people with cancer. In addition, some ncRNAs act on the same target through multiple pathways, and there is a feedback mechanism in many regulatory processes. circRNAs, on the other hand, primarily serve as sponges, being occasionally co-regulated by various ncRNAs to impact downstream pathways, and their mechanism can prove intricate. More exploration into their underlying regulatory mechanisms is necessary, bolstered by the added complexity of the paired ROS interaction. Moreover, a certain state of cancer cells is not only regulated by a specific substance, and it remains unclear whether the changes of downstream pathway molecules interact with each other when ncRNAs act simultaneously. Multi-targeted therapeutic approach may be a promising solution here. Notably, nowadays, cancer drug resistance is frequent, and ncRNAs and oxidative stress have been associated with the modulation of drug resistance pathways in some studies in addition to affecting the efficacy of drug treatments. Therefore, incorporating drug sensitivity assessments in clinical therapy plans might be pivotal.

## Funding

This work was funded by the Hengyang City Science and Technology Planning Project (Hunan, China) (No. 202150063473), the Scientific Research Project of Hunan Provincial Health Commission (China) (No. 202202044140), and the Scientific Research Project of Hunan Provincial Education Department (China) (No. 21B0438).

## Conflict of interests

The authors declared no competing interests.

## Abbreviations

Ang2: angiopoietin-2
AKT: protein kinase B
AP1: activator protein 1
ATG: autophagy-associated gene
Bax: Bcl-2-associated X protein
Bcl: B-cell lymphoma
Bim: Bcl-2-like protein 11
Bmi-1: B-cell-specific Moloney murine leukemia virus integration site 1
BNIP3: BCL2 interacting protein 3
circRNA: circular RNA
CK2α: casein kinase 2a
CTH: cystathionine gamma-lyase
EMT: epithelial–mesenchymal transition
ERK: extracellular signal-regulated kinase
FOXO: forkhead box O
GAS: growth arrest-specific
GLS1: glutaminase 1
GLUT1: glucose transporter 1
GPX4: glutathione peroxidase 4
GSK: glycogen synthase kinase
G6PD: glucose-6-phosphate dehydrogenase
lncRNA: long non-coding RNA
MAPK: mitogen-activated protein kinase
MDM2: murine double minute 2
miRNA: microRNA
MMP: metalloproteinase
MT-ND2: mitochondrially encoded NADH dehydrogenase 2
ncRNA: non-coding RNA
NF-κB: nuclear factor-κB
NIX: Nip-like protein X
NOX: NADPH oxidase
NRF2: nuclear factor erythroid 2-related factor 2
HIF: hypoxia-inducing factor
HK2: hexokinase 2
HOXB7: homeobox B7
HO-1: heme oxygenase-1
HSP: heat shock protein
IGF: insulin-like growth factor
KLF4: kruppel-like factor 4
LDHA: lactate dehydrogenase A
LLGL1/2: lethal giant larval protein homolog 1/2
MEG3: maternally expressed gene 3
MYCC: myelocytomatosis oncogene cellular homolog
PAK2: p21-activated protein kinase
Pax6: paired box 6
PDCD4: programmed cell death protein 4
PFK: phosphofructokinase
Pgc-1α: peroxisome proliferator-activated receptor γ coactivator 1α
PHD2: prolyl hydroxylase domain-containing protein 2
PI3K: phosphatidylinositol-3 kinase
PKM: pyruvate kinase M
PPARγ: peroxisome proliferator-activated receptor γ
PRDX: peroxiredoxin
PTBP: polypyrimidine tract binding protein
PTEN: phosphatase and tensin homolog
PTGR2: prostaglandin reductase 2
Raf: rapidly accelerated fibrosarcoma
Ras: rat sarcoma
ROS: reactive oxygen species
PRC2: polycomb repressive complex 2
RSL3: Ras-selective lethal small molecule 3
RTK: receptor tyrosine kinase
SLC7A11: xCT cystine/glutamate transporter
Smad: mothers against decapentaplegic homolog
SOD: superoxide dismutase
SPRY2: sprouty homolog 2
SRIT: sirtuin
STAT3: signal transducer and activator of transcription 3
TGF: transforming growth factor
TIMMDC1: translocase of inner mitochondrial membrane domain-containing 1
TOR: target of rapamycin
TRIM37: triadic motif family protein 37
VEGF: vascular endothelial growth factor
Wnt: Wingless/Integrated
ZEB1: zinc finger E homology cassette binding 1
α-SMA: α-smooth muscle actin
