# Supplementary material for: The crosstalk between non-coding RNAs and oxidative stress in cancer progression
Source: Genes Dis. 2024 Apr 10;12(3):101286. doi: 10.1016/j.gendis.2024.101286 (PMC11870203; doi:10.1016/j.gendis.2024.101286)
Supplement: Multimedia component 1 [file mmc1.docx]

**Summary**

This manuscript delves into the influence of non-coding RNAs (ncRNAs) and oxidative stress on the existence of cancer cells throughout cancer evolution. In conversation with cancer cell functionality during cancer development, the interplay between ncRNAs and oxidative stress warrants discussion. Given the fragility of cancer genomes hindering conventional therapeutic measures such as medication and chemotherapy, precise treatments serve as a significant advancement towards malignant disease management. Three types of ncRNAs (microRNAs, long non-coding RNAs, and circular RNAs) play a vital part in shaping genetic circuits implicated in cancer cell expansion and invasion. Notably, circular RNAs primarily operate as miRNA sponges due to their constancy, serving competitive binding with messenger RNAs (mRNAs), thus disrupting miRNA-regulated post-transcriptional gene expression. Non-coding RNA regulation of cancer progression is always accompanied by the generation and reduction of reactive oxygen species (ROS), which together with ROS form an inseparable signaling cascade of the cancer regulatory process. Oxidative stress arises from the negative effects of free radicals in the body and is directly related to cancer gene expression and signaling. Within Hanahan's and his esteemed colleagues' comprehensive inquiries, it is revealed that tumor cells exhibit numerous shared attributes. These include, but are not limited to, their capability to circumvent growth inhibitory signals, acquire resistance towards programmed cell death, proliferate incessantly and replicate themselves, manifest genomic instability, manage their energy resources to shield themselves from the host immune system, stimulate neovascularisation to underpin their development, initiate the metastatic cascade and also stimulate inflammation responses intimately linked with tumors. This article investigates the coaction between ncRNAs and oxidative stress in cancerous processes: epithelial-mesenchymal transition (EMT) – representing a signature mode of cancer cell intrusion; cancer cell autophagy – a self-preserving cell suicide phenomenon; angiogenesis – intimately linked to cancer cell proliferation and migration; and energy metabolism – primarily glycolysis, determining the unfolding course of cancer cell life activities. Through in-depth studies, it is established that ncRNAs and oxidative stress have a reciprocal relationship that fuels the growth and spread of tumors. By understanding this correlation, researchers can develop new therapeutic approaches.

EMT is the process by which epithelial cells are converted to a mesenchymal cellular phenotype via a distinctive phenotypic pathway that has been preserved in phylogeny. This intricate biological process involves modifications to cell cycle markers, orchestrated by both intracellular and extracellular stimuli. Specifically, numerous EMT-transcriptional factors (EMT-TFs) such as Snail, Twist1, and ZEB1 play fundamental roles in regulating or influencing this phylogenetically conserved transition. Additionally, non-coding RNAs exert essential influences on this pathway through intricate signaling routes involving WNT/β-catenin, TGF-β/SMAD, TGF-β/non-SMAD, Notch, and others. Notably, these mechanisms are intertwined with oxidative stress and results in reactive oxygen species generation. Evidently, ROS themselves serve as intermediaries in some of these signaling pathways. Within the tumour microenvironment, EMT can be induced and activated by hypoxia, inflammatory cytokines, and mitogenic growth factors, thereby interfering with the efficacy of anti-cancer therapeutic agents. The oxidative stress triggered by hypoxia, predominantly leveraging ROS overproduction, influences the transcription of HIF-1α, with its downstream impacts facilitating cellular adaptation to low oxygen levels. In particular, ncRNAs such as hypoxia-responsive lncRNA (HRL) play critical roles in controlling hypoxic gene expression and manipulating the HIF transcriptional cascade. Hypoxia energizes the mobilization of 'master' EMT transcriptional regulators. zeb1 is a pivotal player in shaping EMT during oncogenesis, influencing EMT functionality and triggering miRNA feedback loops. Also, BNIP3 serves as a versatile cancer suppressor intimately linked with oxidative stress, functioning as a ncRNA target during EMT. Pharmacological interventions focusing on the PI3K/AKT/mTOR pathway in EMT have shown promise in boosting cancer vulnerability and circumventing drug-resistance amongst various types of malignancies.

Autophagy is an evolutionarily conserved catabolic mechanism, innate within cellular systems, that furnishes cells with a steady supply of bio-molecules and energy for maintaining physiological balance under strenuous conditions like the tumour microenvironment. ROS and their inhibitory effects on mitochondrial function may trigger the process of mitochondrial autophagy in a specific way, with a specific pathway revolving around mTORC1. When discussing regulatory mechanisms related to the growth factor signaling pathway in mTORC1, insulin/insulin-like growth factor-1 - phosphoinositide 3-kinase - protein kinase B (PI3K-Akt) emerges as the key avenue. This pathway exerts a negative influence on the initiation of autophagy. Interestingly, autophagy can also be modulated by manipulating autophagy-related genes (ATGs), or through the post-transcriptional regulation of ncRNAs.

The regulation of angiogenesis is directly under the umbrella of various stimuli such as VEGF, Ang, HIF, IGF, and TGF-β, with VEGF emerging as the most significant player in this context. During this intricate targeting process, ROS invariably serve as downstream signal mediators influencing the function of other critical factors or directly impacting angiogenesis which presents multiple levels of regulatory process. Of great interest here are the roles of VEGF-dependent angiogenesis coupled with HIF signaling, a pathway often perturbed by ROS. The interconnection between the HIF1α/ROS pathway initiates tissue-specific angiogenesis via enhanced expression of VEGF, along with its cognate receptors VEGFR1 and VEGFR2. ROS also affects VEGF-induced dimerization and autophosphorylation of VEGFR2, which are indispensable for VEGFR2 activation and consequent angiogenesis. The role of non-coding RNAs in regulating angiogenesis is intriguing; they specifically target the generation of aforementioned angiogenic agonists (cZNF292 notwithstanding).

The energy metabolism permeates virtually every facet of a cancer cell's lifecycle. NcRNAs contribute to the redistribution of tumor metabolism. NcRNAs exhibit a direct influence on the expression patterns of mRNAs and proteins implicated in metabolism, such as PKM, SOD, GLUT1 and G6PD. Additionally, these molecules manipulate the production of pivotal enzymes and associated factors, such as PTBP and c-Myc, thereby impacting diverse metabolic pathways that culminate in the generation of ROS linked to oxidative stress. The production of ROS is inevitable in energy metabolism, and there is a certain correlation between metabolic rate and ROS generation. In particular circumstances, this may induce disturbance in the oxidative status, culminating in cellular oxidative stress. Occasionally, this can induce iron deprivation culminating in cell demise.

Studies have shown that reduced levels of ROS stimulate the expansion and multiplication of malignant cells. In this process, the receptor tyrosine kinase (RTK) signalling process has received extensive attention. Mutations in this signalling pathway often lead to cellular transformation, which is evident in a wide range of malignant tumours and can be regarded as an upstream growth detector. Mutations affect receptor tyrosine kinases or subsequent pathway components such as MAPK, PI3K/AKT, resulting in enhanced cell proliferation, survival, invasiveness and metastatic potential. In terms of pharmacotherapy, metformin associates miR-21 and miR-155 with PI3K/Akt/mTOR, fulfilling the potential role of the mitochondrial pathway to trigger apoptosis to limit tumour growth. In addition, ROS-activated MAPK enhances FOXO function through multiple mechanisms, and oxidative stress induces loss of FOXO activity via the nuclear factor-kappa light chain enhancer of the NF-κB pathway. miR-155 is a prime example of linking this process to the RTK process of cell proliferation. mek/ERK to Bim influences ROS levels, and non-coding RNAs can act as upstream regulators. the occurrence of this process. CircRNAs during cancer cell proliferation always act on ROS regulation related enzymes such as SLC7A11 linked to iron death, and at the same time, she is always a sponge for miRNAs.
